# Baseline and 1-year interim follow-up assessment of Japanese patients initiating insulin therapy who were enrolled in the cardiovascular risk evaluation in people with type 2 diabetes on insulin therapy study: an international, multicenter, observational study

**DOI:** 10.1186/1475-2840-12-131

**Published:** 2013-09-08

**Authors:** Ryuzo Kawamori, Koichi Node, Toshiaki Hanafusa, Yoshihito Atsumi, Yusuke Naito, Yoshitomo Oka

**Affiliations:** 1 Department of Medicine, Metabolism and Endocrinology, Juntendo University Graduate School of Medicine, 2-1-1 Hongo, Bunkyo-ku, Tokyo 113-8421, Japan; 2 Department of Cardiovascular Medicine, Saga University, 5-1-1, Nabeshima, Saga-shi, Saga 849-8501, Japan; 3 Diabetes, Metabolism and Endocrinology, Department of Internal Medicine (I), Osaka Medical College, 2-7, Daigakumachi, Takatsuki-shi, Osaka 569-8686, Japan; 4 Diabetes Center, Eiju General Hospital, 2-23-16, Higashiueno, Taito-ku, Tokyo 110-8645, Japan; 5 Sanofi K.K., Shinjuku-ku, Tokyo Opera City Tower, 3-20-2, Nishi-Shinjuku, Shinjuku-ku, Tokyo 163-1488, Japan; 6 Division of Molecular Metabolism and Diabetes, Tohoku University Graduate School of Medicine, 1-1, Katahira, 2-chome, Aoba-ku, Sendai 980-8577, Japan

**Keywords:** Type 2 diabetes mellitus, Insulin therapy, Real-life clinical data

## Abstract

**Background:**

The Cardiovascular Risk Evaluation in people with type 2 Diabetes on Insulin Therapy (CREDIT) study is an international, multicenter, observational study designed to assess metabolic parameters and cardiovascular risk of patients with type 2 diabetes mellitus (T2DM) on insulin therapy. The present report summarizes results at baseline and 1-year follow-up for the cohort of Japanese patients.

**Methods:**

Male and female patients (n = 511), aged >40 years, with T2DM for >1 year, treated with insulin therapy for ≥1 month and <6 months were eligible for participation in the study. Glycemic and lipid parameters, duration of diabetes, diabetic complications, oral antidiabetic medications, and all hypoglycemic episodes were recorded. Effectiveness was assessed based on changes in clinical parameters and attainment of target HbA1c levels. Safety was evaluated based on episodes of hypoglycemia and weight gain.

**Results:**

At baseline, the mean ± SD duration of diabetes was 11.8 ± 8.8 years. Microvascular and macrovascular diabetic complications were present in 83.4% and 25.1% of patients, respectively. At the 1-year follow-up, significant improvements were observed in mean HbA1c (10.3 ± 2.0% vs. 7.5 ± 1.3%, *P* < .001), fasting plasma glucose (217.3 ± 80.8 mg/dL vs. 139.0 ± 48.7 mg/dL, *P* < .001), and postprandial plasma glucose levels (296.1 ± 96.0 mg/dL vs. 178.2 ± 68.6 mg/dL, *P* < .001) compared with baseline. Mean total cholesterol (*P* < .001), low-density lipoprotein cholesterol (*P* < .001), triglycerides (*P* < .01), and diastolic blood pressure (*P* < .01) also significantly decreased. Good glycemic control (HbA1c < 7.0%) was achieved in 40% of patients at the 1-year follow-up. Glycemic control tended to be better in patients with lower baseline HbA1c levels (*P* < .01). Patients with a shorter duration of diabetes were more likely to achieve glycemic control and discontinue insulin for diabetes control at the 1-year follow-up (*P* < .05 for trend). Symptomatic hypoglycemic episodes occurred in 21.8% of patients over 6 to 12 months.

**Conclusions:**

Our results suggest that insulin treatment is an effective and safe therapeutic option in Japanese patients with T2DM, and earlier insulin initiation might be associated with better glycemic control.

## Background

The incidence of type 2 diabetes mellitus (T2DM) is dramatically increasing worldwide due to increasing obesity, a more sedentary lifestyle, and aging of the population. In 2012, diabetes was estimated to be present in 371 million people worldwide; it is predicted that the prevalence will increase to 552 million by 2030 [1]. Likewise in 2012, the estimated prevalence of diabetes in Japan was 7.1 million people, and this value is expected to increase to 10.2 million by 2030 [1]. Diabetes and its associated complications have become a major cause of morbidity and mortality, representing one of the greatest healthcare challenges facing the world today [2].

Many epidemiologic studies and national registries on diabetes have been conducted at national or regional levels, particularly in developed countries. These studies provide useful information for assessing the current quality of care in diabetic patients and for evaluating compliance with national treatment guidelines [3-9]. However, few studies have focused on diabetes treatment with insulin, one of the most reliable agents for glycemic control. In particular, few data are available regarding the roles of baseline HbA1c levels and the duration of diabetes for predicting future need for insulin therapy. Furthermore, early intervention with insulin in T2DM may prevent disease progression as well as preserve insulin secretion from pancreatic beta cells [10].

The Cardiovascular Risk Evaluation in patients with type 2 Diabetes on Insulin Therapy (CREDIT) study is an international, multicenter (314 centers), non-interventional investigation of the effects of long-term (4 years) glycemic control following initiation of insulin treatment on the risk reduction of cardiovascular (CV) events associated with T2DM in a large cohort of patients (n = 3,031) [11]. This report provides details from the 1-year interim analysis of Japanese patients involved in the CREDIT study, including baseline characteristics at insulin initiation and baseline parameters to predict better glycemic control in real-life settings.

## Methods

### Study design

The CREDIT is a global, multicenter, observational, non-interventional study in medical practice designed to investigate the effects of long-term glycemic control with insulin treatment on the risk reduction of CV events associated with T2DM [11]. The total follow-up period was 4 years, with assessments every 6 months from baseline. The present report details the results for the Japanese cohort of patients at baseline and at the 1-year follow-up.

Participating investigators included randomly selected physicians specializing in diabetes and primary care physicians who are experts in insulin therapy. Each investigator was given a 2-month instruction period with respect to the study protocol, enrolling approximately 10 (range, 5–30) consecutive eligible patients over a 12-month recruitment period. Japanese participants were recruited from January to December 2007. The study was approved by local ethics committees according to the regulations.

### Study population

Male or female patients, aged >40 years, with T2DM for >1 year and who were initiating insulin therapy were eligible. Other inclusion criteria included duration of insulin therapy ≥1 month and <6 months before registration, regardless of the mode of administration, as well as HbA1c levels assessed within 3 months prior to initiation of insulin therapy and an expectation of long-term insulin therapy. Exclusion criteria included type 1 diabetes, non-insulin-treated T2DM, diabetes secondary to pancreatic damage, corticosteroid treatment, endocrine impairment, expectation of short-term insulin therapy (e.g., gestational diabetes, pancreatic cancer, or surgery), participation in another clinical study using insulin, or pregnancy at recruitment.

All patients provided written, informed consent to participate in this study, which was conducted in accordance with the principles of the Declaration of Helsinki and subsequent amendments, and the guidelines for Good Epidemiology Practice.

### Data analysis

The following patient data were recorded at registration: background (gender, age, and disease duration); history of diabetic complications (retinopathy, peripheral neuropathy, nephropathy including microalbuminuria [30–299 mg/24 h], macroalbuminuria [≥300 mg/24 h], and renal failure [confirmed by creatinine clearance], and dialysis and/or transplantation); history of macrovascular disease (i.e., myocardial infarction, stable angina, severe unstable angina leading to hospitalization, heart failure, stroke, transient ischemic attack, peripheral vascular disease, myocardial revascularization, peripheral revascularization, or lower limb amputation); use of oral antidiabetic (OAD) medication at initiation of insulin therapy; and details of insulin therapy at initiation (date, reason, insulin type [e.g., basal, short-acting, premix, others], mode of administration, dosage, and frequency of administration). Any change in insulin therapy during the study was recorded along with the reason for the change.

The following data were recorded at every 6-month visit: systolic blood pressure (SBP), diastolic blood pressure (DBP), body weight, body mass index (BMI), HbA1c levels (HbA1c was collected as the Japan Diabetes Society value and was normalized to 6.0%, regarding the normal upper limit by the center: normalized HbA1c = HbA1c × 6.0/normal upper limit; this method was the standard procedure used in the CREDIT study), fasting plasma glucose (FPG) levels, postprandial plasma glucose (PPG) levels, incidence of symptomatic and severe hypoglycemia, levels of plasma lipids (total cholesterol, high-density lipoprotein (HDL) cholesterol, low-density lipoprotein (LDL) cholesterol, and triglycerides), and serum creatinine levels. Symptomatic hypoglycemia was defined by typical symptoms and plasma glucose levels <70 mg/dL. Severe hypoglycemia was defined as an event with clinical symptoms that were considered to result from hypoglycemia in which the patient required the assistance of another person and had one of the following: plasma glucose level <36 mg/dL or requirement for oral carbohydrate, intravenous glucose, or glucagon administration for resolution. Hypoglycemia was further defined as nocturnal if it occurred during night-time sleep.

Effectiveness was assessed based on changes in the glycemic control index, rate of attaining HbA1c level <7.0%, and rate of withdrawal from insulin therapy. Safety was evaluated based on the rate of hypoglycemia during the 6 months before the 1-year follow-up visit and weight gain.

### Statistical analysis

For baseline characteristics, mean values were expressed with 1 standard deviation (SD) or proportions were shown for categorical variables. The paired *t*-test was used to compare continuous variables at baseline and at 1-year follow-up. Number of patients achieving HbA1c level <7.0%, number of withdrawals at 1-year follow-up, and number of hypoglycemic episodes were tested using the Cochran-Armitage test or trend test using analysis of variance according to baseline HbA1c levels and duration of diabetes. Student’s *t*-test and χ^2^ test (or Fisher’s exact test, if the cell numbers were less) with Hochberg multiple comparison adjustments [12] were used for pairwise comparisons between categories. The level of significance was set at *P* < .05. Data analysis was primarily performed using SAS software version 8.2 (SAS Institute Inc., Cary, NC), with some analysis conducted using R 2.13.1 [13] for Hochberg multiple comparison adjustment.

## Results

A total of 511 patients were recruited by 63 investigators (94.3% of whom were diabetes specialists) at different institutes throughout Japan (63.6% in hospitals and 29.2% at clinics). Insulin administration was started due to poor glycemic control in 87.7% of patients.

Baseline patient characteristics are shown in Table 1. Mean age was 62.2 ± 10.0 years, and approximately two-thirds of patients were men. The mean duration of diabetes was 11.8 ± 8.8 years, and 55.6% had diabetes for ≥10 years. Mean baseline HbA1c level was 10.3 ± 2.0%. Administration of premix insulin was the most frequently used treatment (36.8%). Microvascular diabetic complications were present in 83.4% of patients, and macrovascular diabetic complications were present in 25.1% of patients.

**Table 1:** Baseline patient characteristics

***Parameter***^**1)**^	***No. of patients (categorized data) or mean ± SD (qualitative data)***
Age at insulin initiation, years [N = 511]	62.2 ± 10.0
Gender [N = 511]	
Male	326 (63.8%)
Female	185 (36.2%)
Duration of diabetes, years [N = 504]	11.8 ± 8.8
Duration of diabetes category [N = 504]	
<5 years	99 (19.6%)
≥5, <10 years	125 (24.8%)
≥10, <15 years	134 (26.6%)
≥15 years	146 (29.0%)
HbA1c (%) [N = 506]	10.3 ± 2.0
HbA1c category [N = 506]	
≤8.0%	57 (11.3%)
>8.0%, ≤10.0%	197 (38.9%)
>10.0%, ≤12.0%	169 (33.4%)
>12.0%	83 (16.4%)
FPG (mg/dL) [N = 254]	217.3 ± 80.8
PPG (mg/dL) [N = 329]	296.1 ± 96.0
BMI (kg/m^2^) [N = 490]	23.9 ± 4.0
Insulin regimen	
Basal	32 (6.3%)
Basal + short-acting	134 (26.2%)
Short-acting	130 (25.4%)
Premix	188 (36.8%)
Other	27 (5.3%)
Microvascular diabetic complication [N = 505]	421 (83.4%)
Nephropathy [N = 500]	304 (60.8%)
Microalbuminuria [N = 498]	178 (35.7%)
Macroalbuminuria [N = 505]	185 (36.6%)
Renal failure [N = 509]	78 (15.3%)
Retinopathy [N = 508]	209 (41.1%)
Peripheral neuropathy [N = 509]	265 (52.1%)
Foot ulcer [N = 510]	7 (1.4%)
Macrovascular disease [N = 510]	128 (25.1%)

Abbreviations: *BMI* body mass index, *FPG* fasting plasma glucose, *PPG* postprandial plasma glucose.

^1)^Data are missing for some patients; numbers with available data are shown in parentheses.

Changes in anthropometric, vital, and laboratory data at 1-year follow-up compared with baseline are shown in Table 2. At the 1-year follow-up, significant increases were observed in mean body weight (1.3 ± 4.3 kg, *P* < .001) and BMI (0.5 ± 1.6 kg/m^2^, *P* < .001), and significant decreases were observed in levels of mean HbA1c (−2.7 ± 2.2%, *P* < .001), FPG (−79.0 ± 92.9 mg/dL, *P* < .001), and PPG (−115.9 ± 111.1 mg/dL, *P* < .001). All plasma lipid parameters significantly improved. Mean serum creatinine levels increased significantly (0.11 ± 0.66 mg/dL, *P* < .01). Mean DBP was significantly decreased, whereas no significant changes were seen in mean SBP.

**Table 2:** Anthropometric, vital, and laboratory data at baseline and 1-year follow-up

***Parameter***	***Baseline***		***1-year follow-up***		***Change***		***P-value***^***1)***^
	***n***	***mean ± SD***	***n***	***mean ± SD***	***n***	***mean ± SD***	
Body weight, kg	491	62.2 ± 12.4	434	63.5 ± 11.8	434	1.3 ± 4.3	***
BMI, kg/m^2^	490	23.9 ± 4.0	433	24.4 ± 3.7	433	0.5 ± 1.6	***
HbA1c, %	506	10.3 ± 2.0	482	7.5 ± 1.3	478	−2.7 ± 2.2	***
FPG, mg/dL	254	217.3 ± 80.8	137	139.0 ± 48.7	91	−79.0 ± 92.9	***
PPG, mg/dL	329	296.1 ± 96.0	380	178.2 ± 68.6	256	−115.9 ± 111.1	***
Total cholesterol, mg/dL	439	201.4 ± 43.6	309	187.7 ± 35.9	286	−12.6 ± 44.2	***
LDL-cholesterol, mg/dL	294	122.0 ± 34.0	383	110.5 ± 28.3	239	−11.0 ± 32.0	***
HDL-cholesterol, mg/dL							
Men	277	50.0 ± 13.7	268	53.2 ± 15.2	234	3.1 ± 11.3	***
Women	163	56.8 ± 15.9	151	58.1 ± 15.7	135	2.3 ± 12.1	*
Triglycerides, mg/dL	465	159.7 ± 132.0	447	138.0 ± 85.3	410	−19.7 ± 128.3	**
Creatinine, mg/dL	404	0.76 ± 0.42	422	0.86 ± 0.58	334	0.11 ± 0.66	**
SBP, mmHg	474	132.1 ± 18.5	466	132.8 ± 17.7	435	1.0 ± 20.6	N.S.
DBP, mmHg	474	76.5 ± 11.7	466	74.5 ± 10.8	435	−1.9 ± 12.4	**

Abbreviations: *BMI* body mass index, *DBP* diastolic blood pressure, *FPG* fasting plasma glucose, *Hb* hemoglobin, *HDL* high-density lipoprotein, *LDL* low-density lipoprotein, *PPG* postprandial plasma glucose, *SBP* systolic blood pressure.

^1)^Paired *t*-test.

N.S. not statistically significant, *P < .05, **P < .01, ***P < .001.

The insulin regimen was found to have changed at the 1-year follow-up compared with baseline (Table 3). The use of basal + short-acting insulin and a short-acting insulin alone regimen decreased by approximately 10%, whereas use of a premixed regimen increased by 10%. Approximately 5% of patients were converted to other regimens, whereas the remaining 5% were withdrawn from insulin therapy mainly due to improvement in diabetes control. Altogether 20 withdrawals (74%) were due to improved glycemic control and/or HbA1c <7.0% at 1-year follow-up, while 4 were due to other reasons (1 for hypoglycemia, 1 for prevention of hypoglycemia, 1 for presence of anti-insulin antibodies, and 1 for inability to self-inject insulin), and 3 were due to unspecified reasons.

**Table 3:** Insulin regimen at initiation and at 1-year follow-up

***Insulin regimen***	***At insulin initiation (N = 511)***		***1-year follow-up (N = 488)***	
	***N (%)***	***Mean dose ± SD (Unit)***	***N (%)***	***Mean dose ± SD (Unit)***
Basal	32 (6.3%)	7.2 ± 2.9	35 (7.2%)	11.0 ± 4.5
Basal + short-acting	134 (26.2%)	Basal: 5.0 ± 2.5 Short: 14.9 ± 6.9	84 (17.2%)	Basal: 10.2 ± 8.4 Short: 19.4 ± 8.0
Short-acting	130 (25.4%)	12.8 ± 8.0	54 (11.1%)	17.6 ± 10.3
Premix	188 (36.8%)	12.3 ± 7.3	230 (47.1%)	20.4 ± 11.5
Others	27 (5.3%)	-	58 (11.9%)	-
No insulin	0	-	27 (5.5%)	-

Data regarding use of OADs before insulin initiation, at insulin initiation, and at the 1-year follow-up are summarized in Table 4. Approximately 19% of patients began insulin directly after trying diet and exercise therapy without using OADs. The remaining patients used OADs before insulin initiation, and most used ≥2 OADs concurrently. Sulfonylureas were the most frequently used OAD class before insulin initiation. Approximately 50% of patients stopped OAD medication at the initiation of insulin therapy; results were similar at the 1-year follow-up. There was little change in the percentages of use of each OAD except for thiazolidinediones between the baseline and 1-year follow-up results; the use of thiazolidinediones decreased after 1 year.

**Table 4:** Oral antidiabetic drug use before insulin initiation, at insulin initiation, and at 1-year follow-up

***Oral antidiabetic drug***	***Before insulin initiation (N = 511)***	***At insulin initiation (N = 511)***	***1-year follow-up (N = 488)***
No. of oral antidiabetic drugs, n (%)			
0	98 (19.2%)	262 (51.3%)	229 (46.9%)
1	119 (23.3%)	110 (21.5%)	127 (26.0%)
2	170 (33.3%)	91 (17.8%)	94 (19.3%)
≥3	124 (24.3%)	48 (9.4%)	38 (7.8%)
Drug class, n (%)			
Biguanides	148 (29.0%)	109 (21.3%)	120 (24.6%)
Sulfonylureas	357 (69.9%)	133 (26.0%)	99 (20.3%)
Glinides	28 (5.5%)	10 (2.0%)	78 (16.0%)
Thiazolidinediones	134 (26.2%)	80 (15.7%)	36 (7.4%)
α-glucosidase inhibitor	177 (34.6%)	107 (20.9%)	104 (21.3%)
Others	5 (1.0%)	5 (1.0%)	0

**Figure 1:**
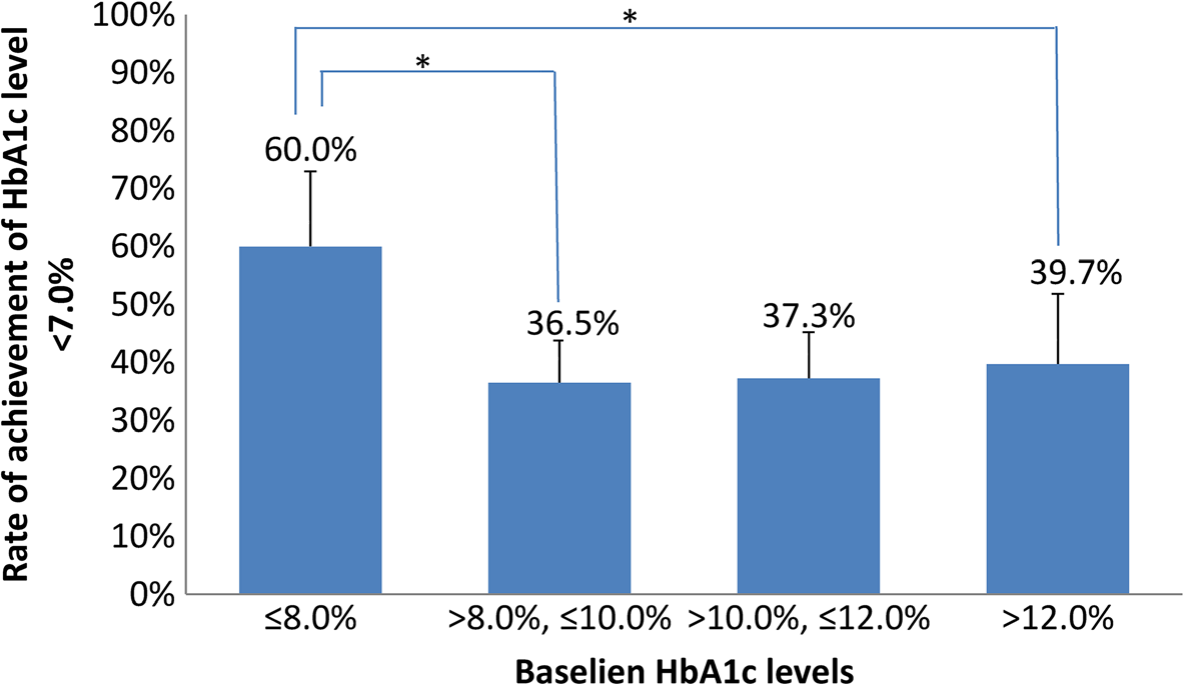
**Percentage of patients achieving HbA1c level <7.0% at the 1-year follow-up compared with baseline.** *P < .05, χ^2^ test with Hochberg’s multiple comparison adjustment.

Good glycemic control was attained in 40% (193/482) of patients at the 1-year follow-up; the glycemic control rate at the 1-year follow-up was significantly higher in patients with a lower baseline HbA1c values (≤8.0%) than in patients with higher baseline HbA1c values (*P* < .01 for Cochran-Armitage test and *P* < .05 for pairwise comparison of ≤8.0% with >8.0%, ≤10.0% or >10.0%, ≤12.0%, Table 5 and Figure 1). Furthermore, 5.5% of patients (27/488) discontinued insulin injections by the time of the 1-year follow-up visit; their mean HbA1c levels had decreased from 10.5 ± 2.1% to 6.8± 1.3% (data not shown). The change in HbA1c from baseline in patients with a shorter duration of diabetes was significantly greater than those with a longer duration (<5 years: -3.5 ± 2.4%; ≥5 years, <10 years: -2.7 ± 2.1%; ≥10 years, <15 years: -2.7 ± 2.3%; and ≥15 years: -2.4 ± 1.9%; P < .01 for <5 years vs. ≥15 years using t test with Hochberg’s multiple comparison adjustment). There was a statistically significant inverse linear trend in the rate of withdrawal from insulin according to duration of diabetes (*P* < .05), but no statistically significant difference was seen between any categorical pairs (Table 6).

**Table 5:** Achieved HbA1c Level <7.0%, withdrawal at 1 year, and hypoglycemic episodes by baseline levels

	**All**^**1)**^	**≤8.0%**	**>8.1%, ≤10.0%**	**>10.0%, ≤12.0%**	**>12.0%**	***P***^**2)**^
Achievement of HbA1c level <7.0%	193/482 (40.0%)	33/55 (60.0%)	69/189 (36.5%)	60/161 (37.3%)	29/73 (39.7%)	**^3)^
Withdrawal from insulin at 1 year	27/488 (5.5%)	5/55 (9.1%)	7/191 (3.7%)	9/164 (5.5%)	6/74 (8.1%)	N.S.
Patients with any hypoglycemic episodes	105/482 (21.8%)	15/54 (27.8%)	39/189 (20.6%)	34/162 (21.0%)	16/73 (21.9%)	N.S.
Number of hypoglycemic episode/patient	1.44 ± 5.18	1.85 ± 6.43	1.13 ± 3.62	1.56 ± 5.19	1.58 ± 7.23	N.S.
Patients with severe hypoglycemic episodes	8/483 (1.7%)	3/54 (5.6%)	3/190 (1.6%)	1/162 (0.6%)	1/73 (1.4%)	*^3)^
Number of severe hypoglycemic episodes/patients	0.03 ± 0.35	0.17 ± 0.97	0.02 ± 0.18	0.01 ± 0.08	0.01 ± 0.12	**

^1)^Including patients with missing baseline HbA1c level data.

^2)^Trend test by Cochran-Armitage test for categorical variables and analysis of variance with contrast statement for numerical variables.

^3)^Category score = (2,1,1,1).

*N.S.* not statistically significant, *P < .05, **P < .01.

**Table 6:** Achieved HbA1c Level <7.0%, withdrawal at 1 year, and hypoglycemic episodes by duration of diabetes

	**All**	**<5 years**	**≥5, <10 years**	**≥10, <15 years**	**≥15 years**	***P***^**1)**^
Achievement of HbA1c level <7.0%	193/482 (40.0%)	44/95 (46.3%)	49/120 (40.8%)	50/129 (38.8%)	47/132 (35.6%)	N.S.
Withdrawal from insulin at 1 year	27/488 (5.5%)	9/95 (9.5%)	8/121 (6.6%)	7/129 (5.4%)	3/137 (2.2%)	*
Patients with any hypoglycemic episodes	105/482 (21.8%)	19/95 (20.0%)	17/120 (14.2%)	39/127 (30.7%)	29/134 (21.6%)	N.S.
Number of any hypoglycemic episodes/patient	1.44 ± 5.18	0.84 ± 2.72	1.06 ± 4.81	2.61 ± 7.87	1.11 ± 3.22	N.S.
Patients with severe hypoglycemic episodes	8/483 (1.7%)	2/95 (2.1%)	1/120 (0.8%)	2/128 (1.6%)	3/134 (2.2%)	N.S.
Number of severe hypoglycemic episodes/patient	0.03 ± 0.35	0.03 ± 0.23	0.01 ± 0.09	0.02 ± 0.12	0.07 ± 0.62	N.S.

^1)^Trend test by Cochran-Armitage test for categorical variables and analysis of variance with contrast statement for numerical variables.

*N.S.* not statistically significant, *P < .05.

Symptomatic hypoglycemic episodes occurred in 105 of 482 (21.8%) patients during 6 to 12 months, and mean frequency of symptomatic hypoglycemia was 1.44 ± 5.18 episodes per patient (nocturnal: 0.04 ± 0.47 episodes per patient). Severe hypoglycemic episodes occurred in 8 of 483 (1.7%) of patients, and the mean frequency of severe hypoglycemia was 0.03 ± 0.35 episodes per patient. There were no cases of severe nocturnal hypoglycemia. A statistically significant inverse linear trend of severe hypoglycemia was observed according to baseline HbA1c levels (*P* < .05 for the number of patients and *P* < .01 for the mean frequency of episodes), but no statistically significant difference was seen between any categorical pairs (Table 5).

## Discussion

The CREDIT Japan interim analyses demonstrated the baseline characteristics of Japanese patients with T2DM who initiated insulin therapy. At baseline, the mean age was 62.2 years, mean disease duration was 11.8 years, and mean HbA1c level was 10.3%. Furthermore, 83.4% had microvascular complications, and 25.1% had macrovascular complications. In addition, the 1-year follow-up results demonstrated that although mean body weight, BMI, and serum creatinine levels increased significantly, 40% of patients treated with insulin achieved good glycemic control, and 5.5% were withdrawn from insulin therapy due to improvement in HbA1c level. There was a higher probability of achieving target HbA1c levels if HbA1c levels at initiation of insulin therapy were lower. The tendency to withdraw from insulin therapy due to improved glycemic control was higher if the duration of diabetes at initiation was shorter.

Few studies have clearly demonstrated the above-mentioned relationship, although some studies have investigated baseline HbA1c levels in order to clarify optimal insulin therapy of T2DM. The following database studies only referred to a snap-shot of mean HbA1c levels in patients who had started insulin therapy. The Swedish National Diabetes Register (N = 61,890) showed that the mean HbA1c level was 7.2–7.9% and that the HbA1c levels were independent of disease duration [4]. The Scottish Care Information-Diabetes Collaboration database registry (N = 36,254) [3] showed that mean HbA1c levels were 8.6–9.4% in type 1 or 2 diabetes patients treated with insulin. The Hong Kong Diabetes Registry (N = 7,549) [8] showed that mean HbA1c levels were 7.9% (insulin alone) or 8.6% (insulin plus OADs).

Compared with previous data at insulin initiation, our results show higher mean baseline HbA1c levels and longer mean diabetes duration. CoDiC® Japanese data showed that insulin therapy was started earlier (mean HbA1c levels, 8–9%) than that in our patients [5,6], and that study concluded that early-stage initiation of insulin therapy was related to satisfactory glycemic control. A large retrospective database study in the United States demonstrated that patients who began intermediate-acting insulin with neutral protamine Hagedorn (NPH) insulin or long-acting insulin with insulin glargine had mean HbA1c values of 8.91% and 9.28%, respectively [7].

In addition to differences in study designs, there are several reasons for this difference. The lack or lower accessibility of long-acting insulin analogues (insulin glargine and detemir) during the enrollment period in Japan may be one of the reasons. These analogues allow a wide range of therapeutic options, from convenient add-on therapy to OADs (basal-supported oral therapy; BOT) to basal-bolus therapy (i.e., basal + short-acting regimen). Currently, BOT is recognized as a convenient and patient-friendly option for insulin initiation. The other reason may be the dominance of in-hospital initiation (63.6%) of insulin therapy; many patients avoid inpatient initiation for various reasons, such as less flexibility to the patient’s lifestyle, leading to delayed initiation. These observations suggest that our cohort potentially represents a more difficult-to-treat and resistant patient population. Further studies are warranted to clarify these details.

Differences in treatment guidelines may also affect the timing of insulin initiation. The consensus statement of the American Diabetes Association (ADA) and the European Association for the Study of Diabetes (EASD) clearly states the goal of glycemic control (HbA1c level < 7.0%) and recommends timely intensification by changing regimens in patients uncontrolled on their current therapy over 3 months [14], though this statement was recently updated in 2012 and addressed individualized rather than uniform treatment [15]. In contrast, Japanese diabetes therapeutic guidelines do not clearly specify the timing or methods for insulin initiation and intensification, although the guidelines recommend achieving goal glycemic values [16].

The results of this study revealed that lower HbA1c levels and a shorter duration of diabetes at insulin initiation were associated with better glycemic control and withdrawal due to HbA1c improvement; hence, earlier intervention is important for successful glycemic control in patients with T2DM. Earlier use of insulin leads to a decreased subsequent disease risk and the preservation of beta cell function. Several clinical trials or systematic review of clinical trials support the results of the present study. The Outcome Reduction with an Initial Glargine Intervention (ORIGIN) trial [17] showed no increase in clinical CV events with insulin glargine for early T2DM after 6.2 years, and a substudy of the ORIGIN, the Glucose Reduction and Atherosclerosis Continuing Evaluation Study (ORIGIN-GRACE) [18] also revealed that long-term treatment with insulin might result in CV event reduction. Hemmingsen et al. [19] summarized that there was no evidence or even towards improved CV mortality with metformin and insulin, compared with insulin alone in T2DM. Furthermore, Holman et al. [20] revealed that intensive intervention immediately after the diagnosis of diabetes leads to decreased CV risk after a long period in the United Kingdom Prospective Diabetes Study (UKPDS). Data from intensive insulin treatment with continuous subcutaneous insulin infusion [10,21] demonstrated several pieces of evidence associated with beta cell preservation and restoration compared with conventional OAD treatment.

In addition to information regarding glycemic control, patients in the present study showed improvement in CV risk factors (lipid profile [22-24] and DBP [25,26]) during the 1-year follow-up. The complete CREDIT 4-year follow-up data will reveal the long-term effects of insulin therapy on CV event reduction.

Several limitations regarding our results must be addressed. First, the CREDIT study was an observational study with no intervention or control group. To increase data reliability compared with previous retrospective or cross-sectional studies, our study was designed as a prospective cohort study. Furthermore, participating investigators were randomly selected to minimize potential bias. In addition, participating investigators were diabetes specialists (94.3%), which likely enhanced data reliability and accuracy. Second, although the grouping of insulins into categories enabled us to include an adequate number of subjects in each insulin group, this method allowed both human and analogue insulins to be included in the same category. Third, the data do not include information on patients treated with long-acting insulin analogues.

## Conclusions

Improvements in both glycemic control as well as lipid parameters at 1-year after insulin initiation in this subgroup analysis of the CREDIT study suggest that insulin treatment is an effective and safe therapeutic option in Japanese patients with T2DM. Additionally, more appropriate timing regarding insulin initiation (i.e., earlier than that used in 2007) may be important for achieving better glycemic control.

## Abbreviations

ADA: American diabetes association; BMI: Body mass index; BOT: Basal-supported oral therapy; CoDiC: Computerized diabetes care; CREDIT: Cardiovascular risk evaluation in people with type 2 diabetes on insulin therapy; CV: Cardiovascular; DBP: Diastolic blood pressure; EASD: European association for the study of diabetes; FPG: Fasting plasma glucose; HDL: High-density lipoprotein; LDL: Low-density lipoprotein; NPH: Neutral protamine hagedorn; OADs: Oral antidiabetic drugs; ORIGIN: Outcome reduction with an initial glargine intervention; PPG: Postprandial plasma glucose; SBP: Systolic blood pressure; SD: Standard deviation; T2DM: Type 2 diabetes mellitus; UKPDS: United Kingdom prospective diabetes study.

## Competing interests

R.K. received an advisory board fee as an advisory board member from Sanofi and received honoraria for lectures from Sanofi, Novo Nordisk, and Eli Lilly. K.N. received an advisory board fee as an advisory board member from Sanofi and is working for an institution participating in this study; a research fee was also received from Sanofi. T.H., Y.A., and Y.O. received advisory board fees as advisory board members from Sanofi. Y.N. is an employee of Sanofi K.K.

## Authors’ contributions

R.K., K.N., T.H., Y.A., Y.O. performed the study. Y.N. conceived of the study, and participated in its design, performed the statistical analysis, and helped draft the manuscript. All authors read and approved the final manuscript.

## Study sites participating in CREDIT study Japan

Aichi Medical University Hospital, Aomori City Hospital, Brain Attack Center, Oota Memorial Hospital, Chubu Rosai Hospital, Fukui Red Cross Hospital, Fukuoka-Higashi Medical Center, Fukuoka Tokushukai Medical Center, Gifu University Hospital, Higami Hospital, Higashiyama Takeda Hospital, Hiroshima General Hospital, Hokkaido University Hospital, Iida Municipal Hospital, Ikeda Hospital, Imamura Hospital Bunin, Japanese Red Cross Medical Center, Jinnouchi Hospital, Juntendo University Hospital, Kagawa University Hospital, Kanazawa Medical University Hospital, Kanazawa University Hospital, Kawashima Hospital, Kido Hospital, Kishiwada City Hospital, Kondo Clinic, Kurashiki Central Hospital, Kurume University School of Medicine, Maebashi Red Cross Hospital, Matsue City Hospital, Matsuyama Shimin Hospital, Meitetsu Hospital, Mimihara General Hospital, Mishuku Hospital, Miyamoto Clinic, Nagano Red Cross Hospital, Nakayama Clinic, Nigata Prefectural Koide Hospital, Nishikawa Hospital, Nishisaitama-chuo National Hospital, Noto General Hospital, Odate Municipal General Hospital, Ohta General Hospital Diabetic Center, Osaka Medical College Hospital, Osaka Prefectural General Hospital, Saga University Hospital, Saitama Red Cross Hospital, Sakakibara Heart institute, Sapporo City General Hospital, Seikeikai Hospital, Shimane University Hospital, Shinkoga Hospital, Shinsyu University Hospital, The Jikei University Aoto Hospital, The Jikei University Kashiwa Hospital, Tokyo Medical University Hospital, Toyama Red Cross Hospital, Toyama University Hospital, Tsunashimakai Kousei Hospital, Wakayama Rousai Hospital, Yamaguchi University Hospital, Yamane Hospital, Yokohama Rosai Hospital, and Yoyogi Hospital.

## References

[B1] 5th Edition of the Diabetes Atlas released on World Diabetes Dayhttp://www.idf.org/diabetesatlas/news/fifth-edition-release

[B2] Zimmet P Alberti KG Shaw J Global and societal implications of the diabetes epidemic Nature 2001 414 782 787 10.1038/414782a 11742409

[B3] Colhoun HM Use of insulin glargine and cancer incidence in Scotland: a study from the Scottish Diabetes Research Network Epidemiology Group Diabetologia 2009 52 1755 1765 10.1007/s00125-009-1453-1 19603149 PMC2723678

[B4] Eliasson B Eeg-Olofsson K Cederholm J Nilsson PM Gudbjornsdottir S Antihyperglycaemic treatment of type 2 diabetes: results from a national diabetes register Diabetes Metab 2007 33 269 276 10.1016/j.diabet.2007.02.003 17499541

[B5] Kobayashi M Yamazaki K Kanatsuka A Investigation of actual conditions concerning insulin therapy in diabetic patients Prog Med 2009 29 1115 1124

[B6] Kobayashi M Yamazaki K Kanatsuka A on behalf of the Japanese Diabetes Clinical Data Management Study Group CoDiC(R): Surveillance of clinical management of diabetes in Japan (2 nd report) Jpn J Diabet Mast Clin 2007 5 401 406

[B7] Rhoads GG Kosiborod M Nesto RW Fonseca VA Lu SE Zhang Q Foody JM Comparison of incidence of acute myocardial infarction in patients with type 2 diabetes mellitus following initiation of neutral protamine Hagedorn insulin versus insulin glargine Am J Cardiol 2009 104 910 916 10.1016/j.amjcard.2009.05.030 19766755

[B8] Tong PC Ko GT So WY Chiang SC Yang X Kong AP Ozaki R Ma RC Cockram CS Chow CC Chan JC Use of anti-diabetic drugs and glycaemic control in type 2 diabetes-tThe Hong Kong Diabetes Registry Diabetes Res Clin Pract 2008 82 346 352 10.1016/j.diabres.2008.09.006 18926583

[B9] Atsumi Y Survey on insulin therapy in patients with diabetes -CANDO study, second report-Shinyaku to Rinsho (J New Rem & Clin) 2010 59 1447 1465

[B10] Weng J Li Y Xu W Shi L Zhang Q Zhu D Hu Y Zhou Z Yan X Tian H Ran X Luo Z Xian J Yan L Li F Zeng L Chen Y Yang L Yan S Liu J Li M Fu Z Cheng H Effect of intensive insulin therapy on beta-cell function and glycaemic control in patients with newly diagnosed type 2 diabetes: a multicentre randomised parallel-group trial Lancet 2008 371 1753 1760 10.1016/S0140-6736(08)60762-X 18502299

[B11] Freemantle N Balkau B Danchin N Wang E Marre M Vespasiani G Kawamori R Home PD Factors influencing initial choice of insulin therapy in a large international non-interventional study of people with type 2 diabetes Diabetes Obes Metab 2012 14 901 909 10.1111/j.1463-1326.2012.01613.x 22519930 PMC3466417

[B12] Hochberg Y A sharper Bonferroni procedure for multiple tests of significance Biometrika 1988 75 800 802 10.1093/biomet/75.4.800

[B13] R Development Core Team R: A Language and Environment for Statistical Computing 2011 Vienna, Austria: R Foundation for Statistical Computing

[B14] Nathan DM Buse JB Davidson MB Ferrannini E Holman RR Sherwin R Zinman B American Diabetes A, European Association for Study of D Medical management of hyperglycemia in type 2 diabetes: a consensus algorithm for the initiation and adjustment of therapy: a consensus statement of the American Diabetes Association and the European Association for the Study of Diabetes Diabetes care 2009 32 193 203 10.2337/dc08-9025 18945920 PMC2606813

[B15] Inzucchi SE Bergenstal RM Buse JB Diamant M Ferrannini E Nauck M Peters AL Tsapas A Wender R Matthews DR Management of hyperglycemia in type 2 diabetes: a patient-centered approach: position statement of the American Diabetes Association (ADA) and the European Association for the Study of Diabetes (EASD) Diabetes care 2012 35 1364 1379 10.2337/dc12-0413 22517736 PMC3357214

[B16] Japan Diabetes Society Treatment Guide for Diabetes 2012 Tokyo: Bunkodo

[B17] Gerstein HC Bosch J Dagenais GR Díaz R Jung H Maggioni AP Pogue J Probstfield J Ramachandran A Riddle MC Rydén LE Yusuf S ORIGIN Trial Investigators Basal insulin and cardiovascular and other outcomes in dysglycemia N Engl J Med 2012 367 319 328 22686416 10.1056/NEJMoa1203858

[B18] Lonn EM Bosch J Diaz R Lopez-Jaramillo P Ramachandran A Hâncu N Hanefeld M Krum H Ryden L Smith S McQueen MJ Dyal L Yusuf S Gerstein HC for the GRACE and ORIGIN Investigators Effect of insulin glargine and n-3FA on carotid intima-media thickness in people with dysglycemia at high risk for cardiovascular events: the glucose reduction and atherosclerosis continuing evaluation study (ORIGIN-GRACE Diabetes care 2013 Epub ahead of print 10.2337/dc12-2129 PMC3747889 23564916

[B19] Hemmingsen B Christensen LL Wetterslev J Vaag A Gluud C Lund SS Almdal T Comparison of metformin and insulin versus insulin alone for type 2 diabetes: systematic review of randomised clinical trials with meta-analyses and trial sequential analyses BMJ 2012 344 e1771 10.1136/bmj.e1771 22517929

[B20] Holman RR Paul SK Bethel MA Matthews DR Neil HAW 10-year follow-up of intensive glucose control in type 2 diabetes N Engl J Med 2008 359 1577 1589 10.1056/NEJMoa0806470 18784090

[B21] Hu Y Li L Xu Y Yu T Tong G Huang H Bi Y Weng J Zhu D Short-term intensive therapy in newly diagnosed type 2 diabetes partially restores both insulin sensitivity and beta-cell function in subjects with long-term remission Diabetes care 2011 34 1848 1853 10.2337/dc10-2105 21680726 PMC3142020

[B22] Aviles-Santa L Sinding J Raskin P Effects of metformin in patients with poorly controlled, insulin-treated type 2 diabetes mellitus. A randomized, double-blind, placebo-controlled trial Ann Intern Med 1999 131 182 188 10.7326/0003-4819-131-3-199908030-00004 10428734

[B23] Hayashi T Kawashima S Nomura H Itoh H Watanabe H Ohrui T Yokote K Sone H Hattori Y Yoshizumi M Ina K Kubota K Japan Cholesterol and Diabetes Mellitus Investigation Group Age, gender, insulin and blood glucose control status alter the risk of ischemic heart disease and stroke among elderly diabetic patients Cardiovasc Diabetol 2011 10 86 10.1186/1475-2840-10-86 21978180 PMC3200162

[B24] Taskinen MR Kuusi T Helve E Nikkila EA Yki-Jarvinen H Insulin therapy induces antiatherogenic changes of serum lipoproteins in noninsulin-dependent diabetes Arteriosclerosis 1988 8 168 177 10.1161/01.ATV.8.2.168 3279941

[B25] UK Prospective Diabetes Study Group Tight blood pressure control and risk of macrovascular and microvascular complications in type 2 diabetes: UKPDS 38. UK Prospective Diabetes Study Group BMJ 1998 317 703 713 10.1136/bmj.317.7160.703 9732337 PMC28659

[B26] Giugliano D Quatraro A Consoli G Minei A Ceriello A De Rosa N D’onofrio F Metformin for obese, insulin-treated diabetic patients: improvement in glycaemic control and reduction of metabolic risk factors Eur J Clin Pharmacol 1993 44 107 112 10.1007/BF00315466 8453955

